# CcpA-Dependent Carbon Catabolite Repression Regulates Fructooligosaccharides Metabolism in *Lactobacillus plantarum*

**DOI:** 10.3389/fmicb.2018.01114

**Published:** 2018-05-29

**Authors:** Chen Chen, Yanqing Lu, Linlin Wang, Haiyan Yu, Huaixiang Tian

**Affiliations:** Department of Food Science and Technology, Shanghai Institute of Technology, Shanghai, China

**Keywords:** *Lactobacillus plantarum*, fructooligosaccharide, catabolite control protein A, carbon catabolite repression, metabolic regulation

## Abstract

Fructooligosaccharides (FOSs) metabolism in *Lactobacillus plantarum* is controlled by two gene clusters, and the global regulator catabolite control protein A (CcpA) may be involved in the regulation. To understand the mechanism, this study focused on the regulation relationships of CcpA toward target genes and the binding effects on the catabolite responsive element (*cre*). First, reverse transcription-PCR analysis of the transcriptional organization of the FOS-related gene clusters showed that they were organized in three independent polycistronic units. Diauxic growth, hierarchical utilization of carbohydrates and repression of FOS-related genes were observed in cultures containing FOS and glucose, suggesting carbon catabolite repression (CCR) control in FOS utilization. Knockout of *ccpA* gene eliminated these phenomena, indicating the principal role of this gene in CCR of FOS metabolism. Furthermore, six potential *cre* sites for CcpA binding were predicted in the regions of putative promoters of the two clusters. Direct binding was confirmed by electrophoretic mobility shift assays *in vitro* and chromatin immunoprecipitation *in vivo.* The results of the above studies suggest that CcpA is a vital regulator of FOS metabolism in *L. plantarum* and that CcpA-dependent CCR regulates FOS metabolism through the direct binding of CcpA toward the *cre* sites in the promoter regions of FOS-related clusters.

## Introduction

Lactobacilli have complex nutritional requirements for fermentable carbohydrates and derive metabolic energy from homofermentative or heterofermentative carbohydrate fermentation (Gänzle and Follador, 2012). The broad ecological distribution and diverse habitats of lactobacilli reflect their metabolic flexibility and suggest that they are able to utilize a wide range of carbohydrates (Kant et al., 2011; Goh and Klaenhammer, 2015). Nevertheless, lactobacilli seldom use different carbon sources simultaneously. Rather, they organize their carbohydrate utilization in a hierarchical manner to achieve maximal growth (Zeng et al., 2017). The presence of a preferred carbon source prevents the utilization of the secondary substrate until the more favorable carbon source is exhausted. This hierarchical phenomenon, which was first described in relation to glucose-lactose diauxie in *Escherichia coli*, has been termed carbon catabolite repression (CCR) (Jankovic and Brückner, 2007; Görke and Stülke, 2008). CCR is important for competitive ability of lactobacilli in natural environments, such as the gastrointestinal tract (GIT). As the selection of the preferred carbon source is a major determining factor of growth rate for the microorganisms in GIT, and is also the competitive success compared with other microorganisms (Görke and Stülke, 2008; Becerra et al., 2015; Cardarelli et al., 2015).

The mechanism of CCR in Gram-positive bacteria involves negative regulation mediated by the catabolite control protein A (CcpA), a protein of the LacI-GalR family (Hueck and Hillen, 1995; Swint-Kruse and Matthews, 2009). It is a pleiotropic regulator involved in many important cellular processes. In the presence of a preferred carbon source, usually glucose, CcpA can effectively bind to its cognate operator sites, termed catabolite responsive elements (*cre*) with the assistance of seryl-phosphorylated histidine phosphocarrier protein (HPr-Ser∼P) (Muscariello et al., 2001; Deutscher et al., 2006; Wu et al., 2015). The consensus sequence of a *cre* site typically has a palindromic nucleotide motif, although some variations in nucleotide composition and length have been reported in recent literatures (Kim and Burne, 2017; Yang et al., 2017). The *cre* site is often located in the promoter region or within open reading frames of the regulated genes and operons (Marciniak et al., 2012). The binding of CcpA to *cre* sequences represses transcription of non-preferred metabolism genes (Fujita, 2009; Cai et al., 2012). CCR is relieved when decreased availability of the preferred carbohydrate results in decreased flux through glycolysis. CCR has been reported in several lactobacilli species, in which it is involved in the regulation of carbon catabolism, aerobic and anaerobic metabolism (Ana et al., 2011; Zotta et al., 2012), stress tolerance (Li et al., 2016), and metabolite production. During these regulatory processes, CcpA plays a central role that inactivation the *ccpA* gene leads to partial or complete relief of CCR (Moye et al., 2014; Zeng et al., 2017).

As one of the important prebiotics, fructooligosaccharides (FOSs) are non-digestible food ingredients that can stimulate the growth and activity of beneficial microbes residing in the GIT (Kaplan and Hutkins, 2000; Goh and Klaenhammer, 2015). Despite it have been widely applied in biotechnological applications and food industry, limited information is available about the regulation of FOS metabolism for specific members of the intestinal microbiota. Goh discovered a diauxic growth pattern when *Lactobacillus paracasei* 1195 was grown on media containing 1% FOS and 0.1% glucose, and predicted that the regulation is controlled by CCR via binding of CcpA to the *cre* site (Goh et al., 2007). Some *cre*-like sequences have also been identified in the operons for FOS utilization in *L. acidophilus* NCFM (Barrangou et al., 2003) and *Bifidobacterium breve* UCC2003 (Ryan et al., 2005). In our previous study, two gene clusters (*sacPTS1* and *sacPTS26*) were found to participate in the metabolism of FOS in *Lactobacillus plantarum* ST-III. A number of potential *cre* sites for CcpA binding have been predicted in the regions of putative promoters of the two clusters (Chen et al., 2015).

To determine whether FOS metabolism in *L. plantarum* is regulated by CcpA-dependent CCR, the present study investigated the growth of *L. plantarum* and its mutant strains in CDM medium containing FOS and limited glucose. Growth profiles, sugar consumption, and gene expression were monitored. The binding of CcpA to the *cre* sites was verified using electrophoretic mobility shift assays (EMSA) *in vitro* and chromatin immunoprecipitation assay (ChIP) *in vivo*. Our findings reveal the hierarchical nature of carbohydrate utilization in *L. plantarum*, and highlight the crucial role of CcpA in controlling FOS utilization.

## Materials and Methods

### Bacterial Strains and Growth Conditions

The bacterial strains, plasmids and primers used in this study and their relevant features are listed in **Table 1** and Supplementary Table 1. *E. coli* strain (DH5α and BL21), used as an intermediate cloning and expression host, was grown in Luria-Bertani (LB) medium at 37°C with aeration at 200 rpm/min. *L. plantarum* ST-III and its *ccpA* mutant strain were routinely cultured in de Man-Rogosa-Sharpe (MRS) broth (Merck, Darmstadt, Germany) at 37°C without agitation. To measure the growth of the wild-type and *ccpA* mutant strains, cells were grown in chemically defined medium (CDM) (Robert et al., 2000; Teusink et al., 2005) supplemented with FOS and glucose solutions (sterilized by passing through a 0.22 μm filter) as the basic carbon sources. The FOS used in this study was a commercial compound supplied by Meiji Seika Kaisha (Tokyo, Japan), comprising 9.8% (w/w) fructosyl-nystose, 37.3% (w/w) 1-kestose, 2.3% (w/w) sucrose, 49.1% (w/w) nystose, and 1.3% (w/w) glucose and fructose. Appropriate antibiotics were added to the media at the following concentrations: 100 μg/mL kanamycin, 50 μg/mL ampicillin, 30 μg/mL chloramphenicol and 250 μg/mL erythromycin for *E. coli*, 10 μg/mL chloramphenicol and 10 or 30 μg/mL (for replica plating) erythromycin for *L. plantarum*.

**Table 1:** Strains and plasmids used in this study.

Strain and plasmid	Relevant feature^a^	Source or reference
**Strains**		
***L. plantarum***		CGMCC 0847
ST-III	Wild-type	
Δ*ccpA* ::cat	Derivative of ST-III containing a *lox66*-P32-*cat-lox71* replacement of *ccpA*	This study
Δ*ccpA*	Derivative of ST-III containing a *lox72* replacement of *ccpA*	This study
409-Flag-ccpA	Derivative of ST-III harboring pSIP409-Flag-ccpA	
***E. coli***		
DH5α	For general gene cloning and plasmid construction	Promega
BL21	For protein expression	Novagen
BL21-ccpA	*E. coli* BL21 (DE3) harboring pET-28a-ccpA	This study
**Plasmid**		
pET-28a(+)	Kana^R^, for cloning and protein expression, included His-tag	Novagen
pET-28-ccpA	Kana^R^, pET-28a(+) with *ccpA* gene cloned into *Nhe*I/*Hin*dØ sites	This study
pNZ5319	Cm^R^, Em^R^; for multiple gene replacements in Gram-positive bacteria	Lambert et al., 2007
pNZ5319-up-down	Cm^R^, Em^R^; pNZ5319 derivative containing homologous regions up and downstream of *ccpA*	This study
pNZ5348	Em^R^; contains *cre* under the control of the lp_1144 promoter	Lambert et al., 2007
pSIP409	Em^R^; for shuttle vector in *E. coli*, *gusA* controlled by P_sppQ_	Sørvig et al., 2005
pSIP409-Flag-ccpA	Em^R^; pSIP409 derivative; *gusA* replaced by Flag-tagged *ccpA*	This study


*^a^Kana^R^, kanamycin resistant; Ap^R^, ampicillin resistant; Cm^R^ chloramphenicol resistant; Em^R^, erythromycin resistant.*

### Growth Analysis, Sampling, and Detection of Sugar Consumption Under Mixed Carbon Sources

Overnight cultures of *L. plantarum* ST-III or its *ccpA* mutant strain were transferred with 2% (v/v) inoculum into 500 mL of CDM containing 0.1% glucose and 0.4% FOS. The cultures were incubated without shaking for 16–18 h at 37°C. During the cells’ growth up to the stationary phase, the samples were withdrawn every 2 h to measure OD_600_ for growth analysis. To quantify sugars consumption and gene expression, culture samples were taken per hour (every 10 min in the diauxic growth period) during fermentation and centrifuged (12,000 × *g*, 4°C, 15 min). The supernatants were collected for residual sugar detection, and the cell pellets were used for RNA isolation.

For quantification of sugar consumption in the cell supernatants, high performance anion exchange chromatography (HPAEC) was carried out on an ICS5000 chromatograph (Dionex, Corp., Sunnyvale, CA, United States) with a CarboPac PA20 anion exchange column (Dionex, Corp., ID 3 mm × 150 mm) and a pulsed amperometric detector (PAD) as described previously (L’homme et al., 2001; Sánchez et al., 2004). After centrifugation (15,000 × *g*, 4°C, 15 min), the supernatant of each sample was filtered through a 0.45 μm nylon filter and then analyzed using a gradient elution procedure with H_2_O-250 mM NaOH as the mobile phase. The column was eluted at a flow rate of 0.5 mL/min and the injection volume of sample was 20 μL. Quantitative analyses were carried out using 5 mg/mL of solutions of the FOS standard set (Wako Pure Chemical Industries, Osaka, Japan), glucose, fructose, and sucrose (Sigma-Aldrich, Co., St. Louis, MO, United States).

### RNA Extraction and RT-qPCR Analysis

Total RNA from *L. plantarum* ST-III cells collected from various conditions was extracted using TRIzol reagent (Invitrogen, Shanghai, China) according to the manufacturer’s instructions. RNA was subjected to RNase-free DNase I digestion and then purified using a PrimeScript RT reagent kit (Takara Bio, Dalian, China). The quantity and quality of total RNA were evaluated using a NanoDrop 2000C (Thermo, Waltham, MA, United States) and agarose gel electrophoresis. RNA preparations were stored at -80°C until use. Total RNA was reverse-transcribed to cDNA with a PrimeScript RT reagent kit (Takara Bio, Dalian, China) following the manufacturer’s instructions.

For RT-qPCR analysis, the generated cDNA was mixed with 0.2 μM gene specific primers (Supplementary Table 1) in a total volume of 25 μL. The PCR cycling conditions were as follows: 95°C for 10 min, followed by 40 cycles of amplification at 95°C for 15 s and 60°C for 30 s. All of the samples were measured in triplicate. Gene expression was normalized by the 2^-ΔΔ^*^C^*^T^ method and the 16S rRNA gene was used as the normalized standard.

### Target Gene Structure Analysis and Prediction of *Cre* Sites

Gene structure analysis of *sacPTS1* and *sacPTS26* clusters (Chen et al., 2015) was performed on *L. plantarum* ST-III grown to early logarithmic phase in CDM medium supplemented with FOS. Total RNA was extracted and reverse-transcribed as described above and then cDNA products were generated as templates for PCR.

Primers (Supplementary Table 1) were designed on the basis of intergenic regions spanning the potential cotranscribed genes of the *sacPTS1* and *sacPTS26* clusters, respectively. As positive controls, each region was amplified with the same primers using chromosomal DNA of *L. plantarum* ST-III as the template. As negative controls, total RNA without the reverse transcription stage was used as the template.

Regulatory Sequence Analysis Tools (RSAT)^1^ was used to analyze the consensus motif of the *cre* sites. They were found by scanning all upstream regions in the genome of *L. plantarum* ST-III based on the profile of binding sites of CcpA in *L. plantarum* WCSF1 using the RegPrecise database^2^. A positional frequency matrix (PFM) was constructed for the collection of binding sites and those in the upstream regions of *sacPTS1* and *sacPTS26* clusters were searched. Scores of candidate sites were defined as the sum of positional nucleotide weights as previously described (Sánchez et al., 2004) and values greater than five were considered as the *cre* sites of binding of CcpA.

### Construction of *ccpA* Mutant

The Cre-*lox*-based mutagenesis system was used for gene deletion of *ccpA* (Lambert et al., 2007). DNA fragments corresponding to the chromosomal regions upstream (1047 bp fragment; primer pairs UpF-UpR) and downstream (1001 bp fragment; primer pairs DownF-DownR) of the *ccpA* gene were amplified by PCR using a proofreading DNA polymerase (CWBIO, Shanghai, China) with chromosomal DNA used as the template. The amplicons were respectively cloned in the *Xho*I and *Eco*53kI restriction sites of suicide vector pNZ5319, and the recombinant mutagenesis vector, pNZ5319-up-down, was electroporated into *L. plantarum* ST-III cells. Candidate double-crossover clones, were selected and confirmed by PCR. Then, the *lox66*-P32-*cat-lox71* cassette was resolved to a single double-mutant *lox72* site through the transient plasmid pNZ5348. A double-crossover *ccpA* mutant without any resistance gene (Δ*ccpA* strain) was acquired and confirmed by DNA sequencing as described previously (Capozzi et al., 2011; Chen et al., 2015).

### Expression and Purification of His_6_-Tagged CcpA Protein

Expression and purification of His_6_-tagged CcpA was performed using the pET-28a(+) vector as previously described (Chen et al., 2014) with some modifications. Briefly, a 1,012 bp sequence of the *ccpA* gene was PCR amplified from *L. plantarum* genomic DNA using the primer pair ccpA-F and ccpA-R, which includes the *Hin*dIII and *Nhe*I sites (Supplementary Table 1). The recombinant plasmid pET-28a-ccpA was constructed as described previously (Chen et al., 2014) and the strain harboring this plasmid was named *E. coil* BL21-ccpA.

The His_6_-tagged CcpA protein expression was induced by addition of 1 mM isopropyl-β-D-thioIsopropyl-β-D-thio-galactoside (IPTG) when OD_600_ reached 0.4 to 0.6 at 37°C. The culture was allowed to grow at 25°C for 8 h. Cells were collected by centrifugation at 6000 × *g* for 10 min, and resuspended in binding buffer (0.2 M sodium phosphate, 5 M NaCl, 1 M imidazole, pH 7.4), then 1 mM PMSF and 0.1 mg/mL lysozyme solution were added, and the cells were then lysed by sonication. The cell debris was removed by centrifugation at 10,000 × *g* for 10 min at 4°C before RNase and DNase were added. The soluble protein was purified by nickel ion affinity chromatography using a Chelating Sepharose Fast Flow column (GE Healthcare, Waukesha, WI, United States) according to the manufacturer’s instructions. The purified protein was desalted and concentrated by Amicon Ultra-0.5 centrifugal filter devices (Millipore, Billerica, MA, United States).

### Electrophoretic Mobility Shift Assay

Double-stranded 200-bp DNA fragments (named P*_sacK_*, P*_pts1_*_-_*_sacA_*, P*_agl4_*, and P*_sacR2_*) containing the putative *cre* sites (Supplementary Table 2), which were located in the four promoter regions of the *sacPTS1* and *sacPTS26* clusters, were amplified by PCR using specific primers, respectively (Supplementary Table 1). Then fluorescent FAM was added to these DNA fragments by PCR reaction with Dpx DNA polymerase (TOLO Biotech, Shanghai, China) using the universal primers M13F-47 (FAM) and M13R-48 (Supplementary Table 1). The FAM-labeled probes were purified using a Wizard^®^ SV Gel and PCR Clean-Up System (Promega, United States) and were quantified with NanoDrop 2000C. A competition assay was performed by addition of 100-fold molar excess of unlabelled probe to the EMSA reaction mixture.

Electrophoretic mobility shift assay was performed in a 20 μL reaction volume that contains 40 ng probe and different amounts of purified His_6_-tagged CcpA proteins, in a reaction buffer of 50 mM Tris-HCl (pH 8.0), 100 mM KCl, 2.5 mM MgCl_2_, 0.2 mM DTT, 2000 ng polydIdC and 10% glycerol. After incubation for 30 min at 30°C, the reaction system was loaded onto 2% agarose gels buffered with 0.5× TBE. The gels were scanned with ImageQuant LAS 4000 mini (GE Healthcare) (Almengor et al., 2007). To verify the specific binding of CcpA to the *cre* sites, each putative *cre* site (generated from RSAT analysis according to the consensus motif) was mutated and named *cre*MUT. The main principle of the mutation was as follows: the defined base in the consensus motif was mutated to the other three bases, and the “W” that represents A or T was mutated to “S,” which represents G or C. Specific primers were then synthesized for PCR amplification, and the amplicons were purified and self-ligated with an Ezmax One-Step Cloning Kit (TOLO Biotech, Shanghai, China) following the manufacturer’s instructions. The *cre*MUT sequences were verified by DNA sequencing, and the probe preparation and EMSA analysis were the same as described above.

### Chromatin Immunoprecipitation Assay

Inducible expression vectors carrying Flag-tagged CcpA in *L. plantarum* ST-III were constructed using the pSIP409 vector as previously described (Chen et al., 2014) with some modifications. Briefly, the *ccpA* gene sequence was amplified by PCR using the Flag-ccpA-409F (containing a Flag-tag on the 5′ terminus) and Flag-ccpA-409R primers (Supplementary Table 1). The purified PCR-products were ligated into the same restriction enzyme digested pSIP409 vector to construct the recombinant plasmid. Then, the constructed plasmid pSIP409-Flag-ccpA was transformed into *L. plantarum* ST-III. For the recombinant strain 409-Flag-ccpA, the cells were induced at an OD_600_ of 0.3 by adding the inducing peptide pheromone IP-673 (synthesized by Invitrogen, Shanghai, China) to a final concentration of 50 ng/mL. The cells were fixed in 1% formaldehyde and quenched by the addition of glycine. Then the cells were washed twice with 5 mM Tris-HCl (pH 8.0) containing 25 mg/mL lysozyme and sheared to an average fragment size of 300 to 500 bp by sonication. After centrifugation, the 5 μL supernatant was retained for use as the input sample, and the rest of the supernatant was incubated with Flag antibodies. Normal Rabbit IgG was set up as a negative control. Immunoprecipitated and input samples were de-crosslinked by incubation in 1× IP elution buffer and 5 M NaCl and 20 mg/mL proteinase K for 65°C at 1.5 h. The purified ChIP products and genomic input DNA were analyzed by qPCR using specific primers (Supplementary Table 1). The enrichment of DNA fragments was analyzed with the input DNA samples serving as controls. All of the samples were measured in triplicate.

### Statistical Analysis

The data shown are representative of at least three independent experiments. Student’s *t*-test was used to determine statistical differences. Differences between samples with a *p*-value ≤ 0.05 were considered to be statistically significant.

## Results

### Analysis of Target Gene Structure and *cre* Consensus

**FIGURE 1:**
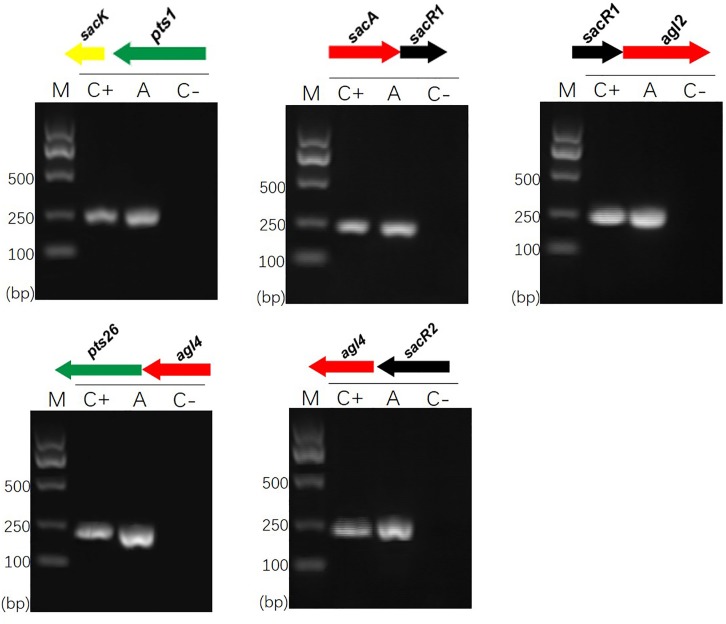
RT-PCR analysis of the transcriptional organization of *sacPTS1* and *sacPTS26* clusters in *L. plantarum* ST-III. M, DNA size marker; C+, positive control using chromosomal DNA of the strain as template; A, fragment amplified with the specific primer set using cDNA generated from FOS-induced *L. plantarum* ST-III cells as a PCR template; C–, negative control using total RNA without the reverse transcription step. Primers used are listed in Supplementary Table 1.

Previous sequence analysis suggested that the FOS-related gene clusters of *L. plantarum* ST-III might be organized in polycistronic units (Chen et al., 2015). To verify this prediction, cDNA products generated from total RNA extracted from cells induced by FOS were used as PCR templates in RT-PCRs to test for the amplification of overlapping regions on each cluster (Vastano et al., 2016). The results of specific amplifications demonstrated that the *sacPTS1* cluster is transcribed in two polycistronic units: *sacK* and *pts1* are transcribed together and *sac*A, *sacR*, and *agl2* are divergently oriented and cotranscribed as an operon. As expected, only a single PCR product was obtained from each amplification reaction for the *sacPTS26* cluster, confirming it constitutes a single operon (**Figure 1**).

**FIGURE 2:**
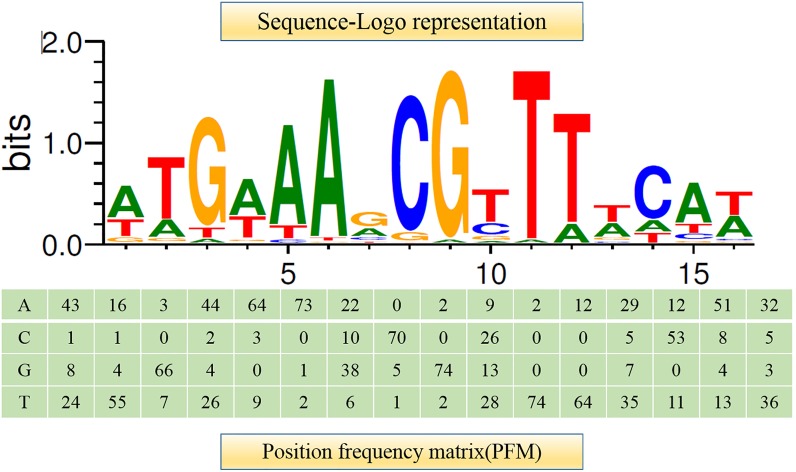
Consensus sequence motif of cre sites in *L. plantarum* ST-III generated using RSAT software. A positional frequency matrix (PFM) was generated according to the occurrence frequency of each base at each location of the consensus sequence. The sequence-logo represents occurrence frequency and the height of each individual symbol reflects its prevalence at a given position.

**FIGURE 3:**
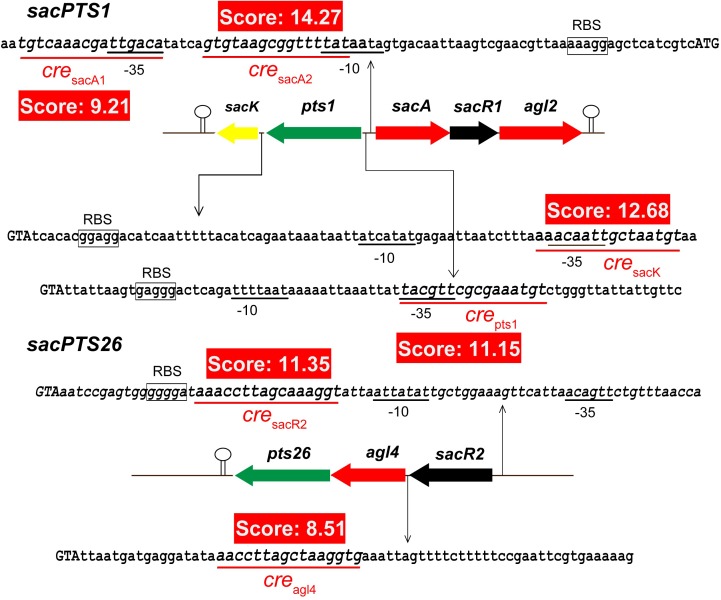
Prediction of the potential *cre* sites in the *sacPTS1* and *sacPTS26* clusters of *L. plantarum* ST-III. Putative *cre* sites are underlined with red; red backgrounds show scores for each *cre* sites, defined as the sum of positional nucleotide weight. The presumed start codon of each gene is shown in capital letters, and the putative-10 and-35 promoter regions and possible ribosome-binding sites (RBSs) are marked.

It has been shown that CcpA can directly bind to *cre* sites to regulate its target genes. However, the *cre* sequence differs among Gram-positive bacteria (Schumacher et al., 2011; Marciniak et al., 2012). This prompted us to search for a conserved common *cre* consensus motif within the genome of *L. plantarum* ST-III based on the RegPrecise database (**Figure 2**). Using the generated PFM to search in the *sacPTS1* and *sacPTS26* clusters, six potential *cre* sites were found. In addition to the five *cre* sites found in the previous study, a new *cre* site was discovered in the P*_pts1_*_-_*_sacA_* region. As a result, this region has three putative *cre* sites (**Figure 3**). The scores of the six candidate sites were all greater than 5, suggesting the possibility of CcpA binding (Medina-Rivera et al., 2011).

### Growth, Sugar Consumption, and Gene Expression of *L. plantarum* ST-III in Medium With Mixed Carbon Sources

**FIGURE 4:**
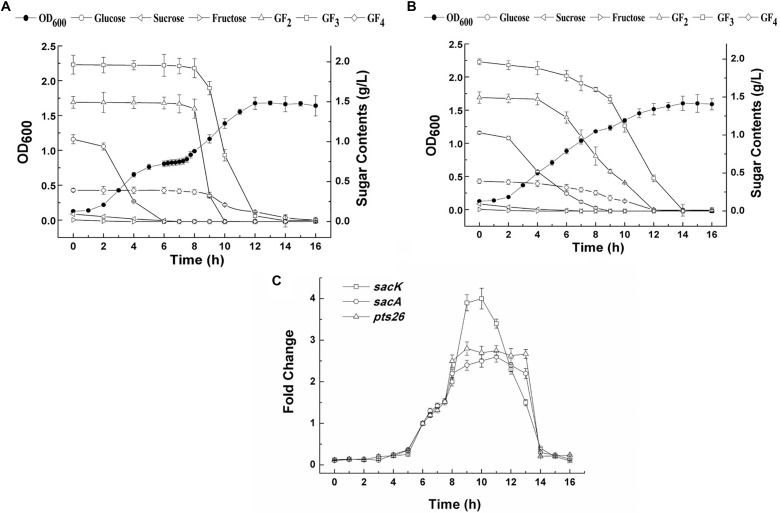
The growth curves, sugar utilization and gene expression of *L. plantarum* ST-III in CDM containing 0.1% glucose and 0.4% FOS. **(A)** The growth curves and sugar consumption of the wild-type strain during fermentation under mixed carbon sources. **(B)** The growth curves and sugar consumption of the Δ*ccpA* strain during fermentation under mixed carbon sources. **(C)** The kinetics of transcription of the FOS-related genes during the diauxic growth of *L. plantarum* ST-III by RT-qPCR. *C*_T_ values obtained at 8 h of incubation were chosen as the control.

The growth of *L. plantarum* ST-III in CDM containing both glucose and FOS (0.1 and 0.4%, respectively) was monitored (**Figure 4A**). A typical diauxic growth pattern with two distinct growth phases separated by a lag phase of about 2 h was observed. During the growth, residual sugars were detected in the supernatant at the same time. In the first 6 h, glucose and traces of fructose in the FOS mixture were consumed and the main components of FOS were kept constant. The diauxic lag is likely to have been caused by the depletion of glucose, as cessation of growth was observed at a similar time. After the diauxic lag phase, the cells resumed growing and entered a second growth phase using FOS as the carbon source. 1-Kestose was rapidly consumed and was undetectable after 10 h of fermentation. Nystose and fructosyl-nystose gradually decreased to an undetectable level at 14 and 16 h, respectively. To examine the kinetics of transcription of the FOS-related genes during the diauxic growth, RT-qPCR was performed to analyze the changes in the expression levels of these genes. RNA samples were collected at different sampling times and *C*_T_ values obtained at 8 h of incubation were chosen as the control values to calculate the fold changes in gene expression over time. Each gene of the two clusters was analyzed using specific primer pairs (Supplementary Table 1). Similar *C*_T_ values were obtained for group of *sacK* and *pts1*, group of *sacA*, *sacR1*, and *agl2* and genes in the *sacPTS26* clusters respectively (data not shown), which is consistent with the result of the RT-PCR experiments. Then *sacK*, *sacA*, and *pts26* genes were chosen as the representatives for gene expression in the subsequent studies. As shown in **Figure 4C**, the expression levels of *sacK* were very low during the first growth phase when glucose was utilized as the preferred carbon source. On entering into the lag phase, gradually higher expression was observed. The maximum transcription levels were observed at 9 and 10 h when 1-kestose and nystose were actively hydrolyzed. The expression profiles of *sacA* and *pts26* were similar. Expression was repressed during the first growth phase, then, after glucose depletion, the repression began to release and the transcription levels remained almost constant during the second growth phase. After 13 h, the transcription levels of all three genes were sharply decreased and this period was associated with the depletion of FOS in the medium. These observations are consistent with the results of previous transcriptome experiments in which glucose repressed the transcription of FOS-induced genes (Denich et al., 2003; Goh et al., 2006).

### *ccpA* Deletion Leads to Relief From CCR

To determine whether the inhibition of FOS utilization by glucose is regulated by catabolic repression via CcpA, a Δ*ccpA* strain was constructed using the Cre-*lox*-based mutagenesis system (Lambert et al., 2007). The growth of the Δ*ccpA* strain on glucose and FOS was compared with its parent strain. The Δ*ccpA* strain exhibited a significantly reduced growth rate compared with the wild-type strain in glucose (a doubling time of 98.4 ± 1.0 min versus 76.2 ± 6 min) and FOS (a doubling time of 102.6 ± 1.5 min versus 81.9 ± 7.2 min). These results are in accordance with findings that CcpA inactivation significantly affected the growth rate of other microorganisms (Cai et al., 2012; Zeng et al., 2017). The deletion of the *ccpA* gene resulted in a complete loss of diauxic growth when the Δ*ccpA* strain was grown in a mixture of glucose and FOS (**Figure 4B**). The components in the FOS mixture were used simultaneously with glucose during the whole growth process, although glucose was consumed at a faster speed. All of the sugars were depleted at the end of fermentation.

Gene expression of the FOS-related clusters in the wild-type and Δ*ccpA* strains was also measured by RT-qPCR in the early logarithmic phase of the growth. The expression levels of the *sacK*, *sacA*, and *pts26* genes in the wild-type and Δ*ccpA* strains were measured with glucose, FOS and the mixture of glucose and FOS as the carbon sources respectively. The *C*_T_ values of the wild-type strain grown on glucose were chosen as the control for the three genes to calculate the fold changes between the different conditions. As expected, all three genes were significantly down-regulated for the wild-type strains grown on glucose and in the mixture of glucose and FOS, compared with the wild-type grown on FOS (**Table 2**). In contrast, after *ccpA* inactivation, repression of these genes by glucose was drastically relieved in the Δ*ccpA* strain compared with the wild-type. For example, the expression levels of the *sacK*, *sacA*, and *sacPTS26* operons in the Δ*ccpA* strain were respectively 2.9-, 3-, and 3.9-fold higher than the corresponding values for the wild-type strain when the cells were grown in a mixture of glucose and FOS. These results confirm the dominant role of CcpA in the CCR of FOS metabolism in *L. plantarum* ST-III. Notably, in the Δ*ccpA* strain, *sacA* and *pts26* showed 1.5- to 1.8-fold increases in the presence of FOS (FOS alone or mixed with glucose) versus glucose alone. This result implies that in addition to the CcpA-dependent CCR, these genes may be induced or derepressed by FOS.

**Table 2:** Relative transcript abundances of FOS-related genes in the wild-type and Δ*ccpA* strains grown in different sugars^a^.

	Wild-type strain			Δ*ccpA* strain		
		
Gene	Glucose	FOS	Glucose+FOS	Glucose	FOS	Glucose+FOS
*sacK^b^*	1 ± 0.37	3.46 ± 0.36^c^	1.17 ± 0.47	3.19 ± 0.13^c^	3.78 ± 0.65^c^	3.38 ± 0.59^c^
*sacA^b^*	1 ± 0.67	3.54 ± 0.04^c^	1.19 ± 0.23	2.15 ± 0.26^c^	3.79 ± 0.08^c^	3.57 ± 0.22^c^
*sacPTS26^b^*	1 ± 0.29	3.46 ± 0.27^c^	0.98 ± 0.09	2.27 ± 0.28^c^	3.31 ± 0.35^c^	3.87 ± 0.26^c^


*^a^Data presented are mean values based on at least three replicates. Error bars indicate standard deviations. ^b^The relative transcription abundances of each gene in different conditions were calculated by comparing of the *C*_T_ values with the values obtained for the wild-type strain grown on glucose. ^c^Statistically significant differences (*p* ≤ 0.05) from values of the wild-type grown on glucose.*

### CcpA Binds to *cre* Motif

**FIGURE 5:**
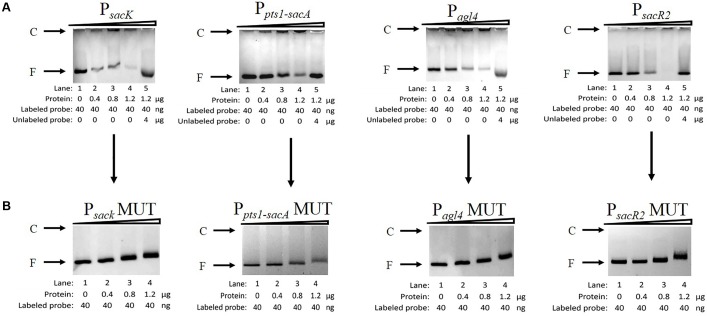
Characterization and verification of the CcpA-DNA binding of the four promoter regions by EMSA. **(A)** EMSA of His_6_-tagged CcpA with the DNA fragments of four promoter regions carrying intact *cre* sites. **(B)** EMSA of His_6_-tagged CcpA with the DNA fragments of four promoter regions carrying mutated *cre* sites. The positions of CcpA-DNA complex (C) or free DNA (F) are indicated to the left of the figure.

To determine whether CcpA can bind to the six putative *cre* sites *in vitro*, His_6_-tagged CcpA was expressed heterologously in *E. coli*, and the purified protein was used to perform an EMSA (Almengor et al., 2007; Tiffert et al., 2008). As shown in **Figure 5A** (lanes 1 to 4), with increasing amounts of His_6_-tagged CcpA (0 to 1.2 μg), the intensities of the bands representing the shifted CcpA-DNA complex strengthened. In contrast, when labeled and unlabelled probes were used for a specific competitive assay (lane 5), no shift was detected for the labeled probe, demonstrating the binding specificity of CcpA to these DNA fragments.

Next, to verify that CcpA combined directly with the *cre* sites within the regions, EMSA experiments were performed to examine whether mutation of *cre* (Supplementary Table 2) affected the interaction between His_6_-tagged CcpA and its DNA fragments (Tiffert et al., 2008; Wu et al., 2015). Then DNA fragments of the four promoter regions, which contained the *cre*MUT sites, were generated by PCR and used in EMSA (Willenborg et al., 2014). The results showed that the binding of His_6_-tagged CcpA protein to the new P*_sacK_*, P_*agl*4_, and P_*sacR*2_ regions was completely abolished (**Figure 5B**), indicating that the CcpA did not bind to the three promoter regions that included *cre*MUT. As the P_*pts*1-*sacA*_ region exists at three putative *cre* sites, the region was mutated three times and EMSA was conducted. The binding affinities of His_6_-tagged CcpA protein to the P_*pts*1-*sacA*_ region were weakened after one (*cre*_sacA1_) or two sites (*cre*_sacA1_ and *cre*_sacA1-sacpts1_) were mutated (data not shown). When the P_*pts*1-*sacA*_ region was mutated at third time (*cre*_sacA2_), the binding affinity of His_6_-tagged CcpA protein to the P_*pts*1-*sacA*_ region vanished. These results indicate that all three *cre* sites can bind to the CcpA protein.

**FIGURE 6:**
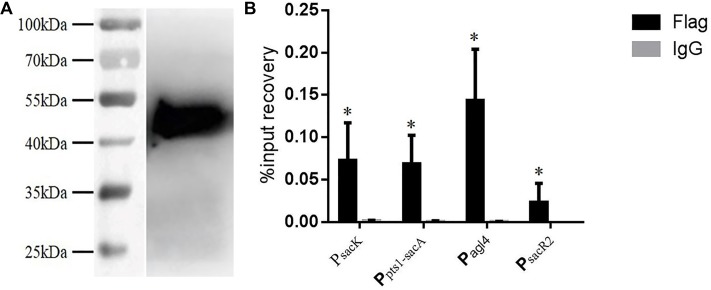
Chromatin immunoprecipitation (ChIP) asssy for analysis of binding of CcpA with the DNA fragment of the four promoter regions. **(A)** Detection of Flag-tagged CcpA protein by western blotting assay with Flag antibodies. **(B)** Enrichment of Flag-tagged CcpA at the promoter regions was determined by ChIP-qPCR. Data presented are mean values based on at least three replicates. Error bars indicate standard deviations. Values that implied statistically significant differences (*p* ≤ 0.05) from negative control (Normal Rabbit IgG) are indicated with asterisks.

Chromatin immunoprecipitation was then performed to test for the binding of CcpA to the promoter regions *in vivo*. The CcpA protein was Flag-tagged at its N terminus, and then the successful expression of 409-Flag-ccpA in *L. plantarum* was confirmed via western blot analysis (**Figure 6A**). The cross-linked DNA fragments were analyzed by RT-qPCR. As shown in **Figure 6B**, it is clear that the four regions were remarkably enriched by CcpA protein compared to the negative control sample, suggesting that CcpA interacts specifically with those promoter regions *in vivo*. Together, these findings suggest that CcpA protein can specifically bind to the four promoter regions, and six *cre* sites exist in these regions with high-affinity binding for CcpA.

## Discussion

*Lactobacillus plantarum* is a normal inhabitant of the human GIT and previous reports have shown that it can utilize FOS as efficiently as glucose, with two gene clusters participating in FOS metabolism (Chen et al., 2015). Due to the complex interspecies competition in the GIT, the regulation of carbohydrate metabolism is crucial to the survival and colonization of *L. plantarum*. CCR is one of the most important regulatory mechanisms used by both Gram-positive and Gram-negative bacteria to achieve maximum growth in mixed carbohydrate environments (Marasco et al., 2002; Almengor et al., 2007; Zeng et al., 2017). In the present work, the FOS metabolism of *L. plantarum* ST-III regulated by CcpA-dependent CCR was studied *in vivo* and *in vitro*.

When *L. plantarum* ST-III was grown in media that contained glucose in addition to FOS, diauxic growth was observed (Cai et al., 2012). Diauxic growth is common among bacteria and higher organisms because most of them preferentially use the carbon sources that are most easily accessible and allow the fastest growth. The presence of preferred carbon sources prevents the expression of catabolic systems that enable the use of secondary substrates (Görke and Stülke, 2008). When the preferred carbon source is exhausted, bacteria first need to synthesize the related enzymes (during the lag phase) before they can resume growth (Kearns and Russell, 1996). Goh also reported this phenomenon when cells were grown on FOS together with limited glucose (Yong, 2005). However, this is disadvantageous for completely fermenting all available sugars and efficiently transforming biomass to high value-added products for microbes in complex carbohydrate environments (Chu, 2015).

The diauxic growth and repression of FOS-related genes were observed in cultures containing glucose and FOS, which suggests that FOS utilization in *L. plantarum* is controlled by CCR. CcpA is believed to play a key role in CCR regulation in Gram-positive bacteria. As expected, in-frame deletion of *ccpA* resulted in the complete loss of diauxic growth, simultaneous use of both sugars and relief of the repressed gene expression. These phenomena are in accordance with previous reports that mutation of the *ccpA* gene leads to complete or partial release from CCR (Marasco et al., 2002; Fujita, 2009). These results show that CcpA is crucial in the CCR of FOS metabolism in *L. plantarum*.

Regulation of the transcription of CcpA-regulated genes involves the binding of CcpA to *cre* sites of the target genes. The consensus sequence of *cre* has been determined as a typical 14- to 16-nucleotides sequence, such as “TGWAANCGNTNWCA” (where W is A or T, N is any base) and “WTGNNARCGNWWWCAW” in *Bacillus subtilis* (Yang et al., 2017) and *L. plantarum* (Wels et al., 2011), respectively. However, it has recently been found that CcpA has flexible binding site architecture that is highly variable in both length and base composition (Yang et al., 2017). In this study, six putative *cre* sites were found in the promoter regions of FOS-related clusters based on the consensus motif generated from RSAT analysis. These sites deviate by one or two nucleotides from published *cre* consensus sequences (Hueck et al., 1994; Miwa et al., 2000; Jankovic and Brückner, 2007), but the binding was verified by EMSA *in vivo* and ChIP *in vitro*. These results provide a new insight into the structure of CcpA recognition sites in Gram-positive bacteria. In addition, the repressor role played by CcpA observed in this study is consistent with the location of *cre* sites within the promoter region. According to Zomer et al. (2007), the repression will occur at *cre* sites located in or downstream of the putative-35 and -10 sequences).

In the absence of a functional CcpA, other regulatory processes could be studied when CcpA-mediated repression is abolished. In our study, FOS in the medium resulted in activation of the FOS-related genes in the Δ*ccpA* strain, demonstrating that other regulators participate in FOS metabolism of *L. plantarum*. These results confirm our previous prediction that *L. plantarum* have a double effect of global and local regulation of FOS metabolism. The local regulators SacR1 and SacR2 may also be involved in metabolic regulation in *L. plantarum* (Chen et al., 2015). In these two clusters, the local regulators SacR1 and SacR2, which are also members of LacI-GalR family, are cotranscribed with other FOS-related genes. In the presence of FOS, SacR1 and SacR2 may be induced by their substrates and maintain their own expression at a certain level to help bacteria to adjust sugar utilization to their metabolic capacities (Robert et al., 2008; Teixeira et al., 2013). Although the effects of CcpA have been confirmed, the interactions between specific local operators and the putative binding sites in these clusters are not directly proven and the exact binding site is not yet clear. To answer these questions, related experiments are currently being carried out.

## Conclusion

The diauxic growth, hierarchical use of sugars and repression of FOS-related genes in *L. plantarum* grown on FOS in the presence of limiting glucose demonstrated that FOS utilization is subject to CCR. Inactivation of the *ccpA* gene eliminated these phenomena, proving the dominant role of CcpA in CCR for FOS metabolism. The binding of CcpA to the *cre* sites in the promoter regions was verified both *in vivo* and *in vitro*, which suggests that CcpA regulates CCR through direct regulation of the transcription of FOS-related clusters. As CCR is part of the regulatory network, further analysis of CCR may lead to deeper insights into a complex regulatory network. Our evolving understanding of the mechanistic interactions of probiotics and prebiotics will provide the molecular basis for the design of effective probiotic–prebiotic combinations to maximize host benefits.

## Author Contributions

CC wrote the manuscript and the statistical analysis. YL analyzed the growth and expression of related genes in wild-type and mutated strains. LW executed the construction of the expression vector and Δ*ccpA* strain. HY executed the target genes structure analysis and confirmed CcpA binding to the putative *cre* sites. HT designed the research.

## Conflict of Interest Statement

The authors declare that the research was conducted in the absence of any commercial or financial relationships that could be construed as a potential conflict of interest.

## Footnotes

1 http://www.rsat.fr/

2 http://regprecise.lbl.gov
