# Supplementary material for: CcpA-Dependent Carbon Catabolite Repression Regulates Fructooligosaccharides Metabolism in Lactobacillus plantarum
Source: Front Microbiol. 2018 May 29;9:1114. doi: 10.3389/fmicb.2018.01114 (PMC5986886; doi:10.3389/fmicb.2018.01114)
Supplement: Supplementary file 1 [file Table_1.PDF]

TABLE 1 Primers used in this study

Primer	Sequence (5' to 3')
**Primers for gene cloning**	
ccpAF	CTAGCTAGCGGCTATTTTATATGGAAAAACAAAC
ccpAR	CGAACTTAATCAGCAGACTTGG
**Primers for gene mutation**	
UpF	CCGCTCGAGATAAAAATAGCCCTTTCTTGCTTG
UpR	GGCTTCCAGACAGACGTCAAAG
DownF	GATATCTGAAAGACTTTCCCACGTTTAGC
DownR	GATATCGTTCGGTTGTCTGCCAGCTAGT
85	GCATTTTGTGAAGTTTTTTTCT
87	CGACTGTACTTTCGGATCCT
108	TGGTTACCAGCCATAATCGAAG
109	GATTTCAAGCACTAGAACCAAG
120	AGAACAATCAAAGCGAGAATAAGG
20	AATAGTTATCTATTATTTAACGGGAGG
CmF	TCTTAGTGACAAGGGTGATAAACTC
CmR	CCGAACCATTATATTTCTCTACATC
EmF	CGATACCGTTTACGAAATTGG
EmR	CTTGCTCATAAGTAACGGTAC
sacK1F	TTTAATGGTCGCACTCATACTGAAC
sacK1R	CCAGCAGCTAAACCTTCTAAACAG
sacAF	GGATGGACACGGATAATCACATT
sacAR	TTGAGAAATAAACCAAGATAGCACC
sacPTS2F	TTAACCAGTGGGATAGGTGCTG
sacPTS2R	CGAGACGACCGTATAAATCAGC
16SF	CGCAAGGCTGAAACTCAAAGG
16SR	CTGACGACAACCATGCACCAC
**Primers for target analysis and EMSA**	
sacKF	CAGCTTCAATTGCACCTAAAAGC
pts1R	AACGACTCAAAGTCACAAATGTCC
pts1F	TAAGTACGCGGTCGGCAACT
sacAR	AGGGGTATAACGGGTTTTACGAT
sacAF	CAATCAGAGCATACTGGAGTCGAT
agl4F	CAACCGGGGTAACATTTGGAT
sacR2R	GGCGGTCGATCAGCTATTACAT
sacR2pF	CGCCAGGGTTTTCCCAGTCACGACAACACTAGGCCGA TGGTTTAAAT
sacR2pR	AGCGGATAACAATTTCACACAGGAGACTAAATGTTTT AAGGGCAAACG
sacKmutF	AAGATTGACCGGGCGCGGAAATTTCTAATTAAGAGTA TACTATT

pts1mutR	GCCCGGTCAATCTTTTAACTTTGACGTAAATGTTGCGT
pts1mutF	AAGATTTGACGGGAGGCGGCTATTAAATTAAAAATAA TTTTAGAC
sacAmutR	CCTCCCGTCAAATCTTGACCCAATAATAACAAGTTTGG AAA
pts1mut2F	CGCAACCATCCCATGATGATATGTCAATCGTTTGACAT
sacAmut2R	TTCGAATCAGCCATCCGCTATCAGTGTAAGCGGTTTTA TAA
pts1mut3F	TCATGGGATGGTTGCGAATAGTGACAATTAAGTCGAA CGT
sacAmut3R	CGCAACCATCCCATGATGATACCTACCGACTAAGCTTT T
agl4mutF	TACGGTTACCGTAAGGGGGATATAGGAGTAGTAATTAT GCAAAA
sacR2mutR	CCTTACGGTAACCGTAGGCTTTAATCAAAAGAAAAAG GC
sacR2pmutF	AATGTGTCAGTCGGAATAGGGGGGTGAGCCTAAAT
sacR2pmutR	CCGACTGACACATTGGGTAATTAATATAACGACCTTTC AAG
M13F-47 (FAM)	CGCCAGGGTTTTCCCAGTCACGAC
M13R-48	AGCGGATAACAATTTCACACAGGA
**Primers for ChIP**	
Flag-ccpAF	CATGCCATGGACGATTACAAGGATGACGACGATAAGG GCATGGAAAAACAAACAGTAAC
Flag-ccpAR	CCGCTCGAGCGAACTTAATCAGCAGACTTGGT
403F	GAAATACCCGTCTAAGGAATTG
403R	TGGTCATGAATTAGTCTCGGA
ChIP-sacKF	AACGATCTGACAGGTTTAATCAGGC
ChIP-pts1R	TACCACCAGCTTCAATTGCACCTA
ChIP-pts1F	AATTATTTTTAATTTAATAATGCAAGCGC
ChIP-sacAR	CGTTCGACTTAATTGTCACTATTATAAAACC
ChIP-agl4F	TAAGCTTAGACTTAATCAAAAGAAAAAGTGC
ChIP-sacR2R	TGCCATCGTTATTAGCATCTTTAAATG
ChIP-PTS26PF	GTGAATTCATTTTGAAAAACCAATTTG
ChIP-PTS26PR	AAACACTAGGCCGATGGTTTAAATAC
