# Supplementary material for: CcpA-Dependent Carbon Catabolite Repression Regulates Fructooligosaccharides Metabolism in Lactobacillus plantarum
Source: Front Microbiol. 2018 May 29;9:1114. doi: 10.3389/fmicb.2018.01114 (PMC5986886; doi:10.3389/fmicb.2018.01114)
Supplement: Supplementary file 2 [file Table_2.PDF]

TABLE 2 Nucleotide sequences of oligonucleotides harbouring the putative *cre* and mutated sites used for EMSA.

cre	Sequence	Mutated *cre*	Mutated sequence[a]
cre$_{sacK}$	TGTAATCGTTAACAAA	*cre*$_{sacK}$MUT	**AAG**A**T**T**GACCGGGCGG**
cre$_{pts1}$	TGTAAAGCGCTTGCAT	*cre*$_{pts1}$MUT	**AAG**A**TTTGA**C**GGGAGG**
cre$_{sacA1}$	TGTCAAACGATTGACA	*cre*$_{sacA1}$MUT	**AAG**C**TT**A**GTCGGT**A**GG**
cre$_{sacA2}$	GTGTAAGCGGTTTTAT	*cre*$_{sacA2}$MUT	**TCA**T**GGGAT**G**GT**T**GCG**
cre$_{agl4}$	GTGGAATCGATTCCAA	*cre*$_{agl4}$MUT	**TAC**G**GT**T**ACCG**T**AAGG**
cre$_{sacPTS26}$	TGGAAACGATTCCAAA	*cre*$_{sacPTS26}$MUT	**AATGTGTC**A**G**TC**GGGG**

[a] Mutated nucleotide are marked with bold.
